# TUG1: a potential endogenous reference gene for long noncoding RNA quantification in blood-based studies

**DOI:** 10.1186/s40364-025-00871-2

**Published:** 2025-12-30

**Authors:** Carlos Rodríguez-Muñoz, Anna Vila, Sally Santisteve, Anna Sánchez-Cucó, Iván D. Benítez, María C. García-Hidalgo, Marta Molinero, Manel Perez-Pons, Anna Moncusí-Moix, Ferran Barbé, Jessica González, David de Gonzalo-Calvo

**Affiliations:** 1 Translational Research in Respiratory Medicine, University Hospital Arnau de Vilanova and Santa Maria, IRBLleida, Avda, Alcalde Rovira Roure 80, Lleida, 25198 Spain; 2 CIBER of Respiratory Diseases (CIBERES), Institute of Health Carlos III, Madrid, Spain; 3 Department of Basic Medical Sciences, Lleida Biomedical Research Institute (IRBLleida), University of Lleida, Lleida, Spain

**Keywords:** Long noncoding RNAs, TUG1, Whole blood, Peripheral blood, Endogenous control, Normalization, Reference genes, Stability, Quantitative real-time polymerase chain reaction

## Abstract

Long noncoding RNAs (lncRNAs) are promising biomarkers, but their accurate quantification by qPCR requires stable endogenous controls. In the present study, we aimed to identify suitable reference lncRNAs for normalization in whole-blood samples. We profiled the expression of 84 lncRNAs and eight commonly used mRNA reference genes by RT-qPCR in samples from 182 individuals. Transcript stability was assessed using three widely applied algorithms: geNorm, NormFinder and BestKeeper. Twenty-nine lncRNAs met predefined expression criteria: consistent detection across all samples, a maximum quantification cycle (Cq) below 33 and a median Cq below 30. Among these, TUG1 consistently ranked as the most stable candidate across all algorithms. FGD5–AS1 and ZFAS1 were also selected based on high stability rankings. We next evaluated the effectiveness of different normalization strategies, comparing them to the gold-standard method, i.e. mean-centering. We included both the selected lncRNAs and eight commonly used mRNA reference genes. TUG1 alone achieved a reduction in expression variability comparable to mean-centering and superior to all other tested strategies, including combinations of multiple reference lncRNAs and mRNAs. Moreover, TUG1 expression showed a minimal association with clinical variables and outcomes. TUG1 was consistently identified among the lncRNAs with the highest expression levels and lowest variability across four independent external RNA-seq datasets. Our findings identify TUG1 as the most stable candidate in this cohort and suggest that it may represent a promising and cost-effective endogenous control for lncRNA quantification in whole-blood studies.

**Supplementary information:**

The online version contains supplementary material available at 10.1186/s40364-025-00871-2.

**To the editor,**

Long noncoding RNAs (lncRNAs) have emerged as promising biomarkers for clinical use [1]. Their regulatory role and disease specificity, combined with their detectability through non-invasive sampling, make them attractive candidates for clinical applications [2]. Furthermore, lncRNAs could be reliably measured in blood-based samples, providing a minimally invasive platform for biomarker discovery and routine implementation [3].

Quantitative real-time polymerase chain reaction (qPCR) is the preferred method for lncRNA quantification [4]. However, technical variability inherent to RNA workflows may arise at multiple stages, including sample collection, isolation, freeze–thaw cycles, RNA extraction, input amount and enzymatic efficiencies [5]. Proper data normalization is therefore essential to minimize technical noise and reveal true biological variation [6]. Traditionally, messenger RNAs (mRNAs) have been commonly used as reference controls for normalization. However, this approach warrants reconsideration, as mRNAs differ from lncRNAs in both biochemical properties and expression dynamics. The use of endogenous controls from the same RNA class is more appropriate. In this context, we conducted a study to identify robust reference lncRNAs for normalization in whole-blood samples using a clinically homogeneous population. This targeted approach provides a controlled framework for evaluating candidate lncRNAs and generates findings that may inform future studies in broader settings.

To accomplish this, we profiled 84 lncRNAs (Supplemental Table S1) using RT-qPCR in a cohort of 182 survivors of COVID-19–induced acute respiratory distress syndrome (ARDS). Of these, 180 samples had complete quantification data and were included in the subsequent analysis (Supplemental Table S2). Details of experimental procedures and data analysis are provided in the Supplemental Material.

Twenty-nine lncRNAs met predefined expression criteria [detected in all samples, with a maximum quantification cycle (Cq) < 33 and a median Cq < 30] and were included in the stability analysis. The remaining 55 were excluded (Supplemental Table S3). Stability was assessed with the three most commonly used algorithms for reference gene validation [7]: geNorm, NormFinder and BestKeeper [8–10]. Table 1 summarizes stability values and rankings. The top candidates were the TUG1/FGD5–AS1 pair (geNorm) and TUG1 (NormFinder and BestKeeper) (Fig. 1A). Since multiple reference genes are generally recommended for accurate normalization [11], we applied a “top-three-in-at-least-two-algorithms” criterion, which resulted in the inclusion of ZFAS1. Thus, TUG1, FGD5–AS1 and ZFAS1 were selected as the final candidate set.

**Fig. 1:**
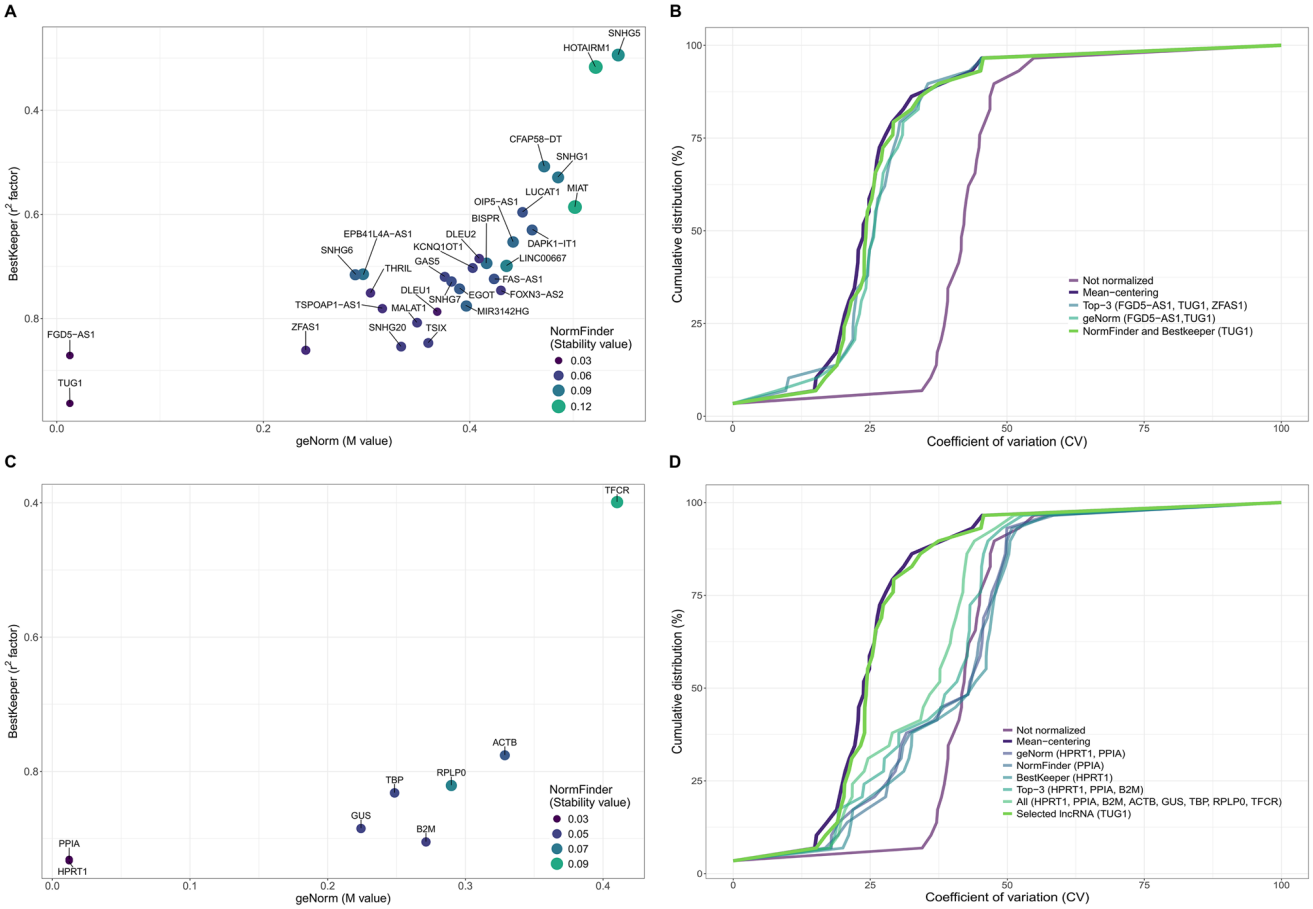
Stability and suitability of candidate endogenous reference long noncoding RNA (lncRNA) and messenger RNAs (mRNAs). **A**) Stability values for each candidate lncRNA calculated using geNorm, NormFinder and BestKeeper. **B**) Cumulative distribution plot of coefficient of variation (CV) values for lncRNA expression data under various conditions: not normalized, mean-centering, best-ranked lncRNA from each algorithm and the top three lncRnas ranked in at least two algorithms. **C**) Stability values for each candidate mRNA calculated using geNorm, NormFinder and BestKeeper. **D**) Cumulative distribution plot of CV values under different conditions: not normalized, mean-centering, best-ranked mRNA from each algorithm, top three mRNAs ranked in at least two algorithms, all eight mRNAs combined and the selected lncRNA (TUG1)

**Table 1:** Expression levels, stability values, and rankings from independent stability analyses for candidate endogenous long noncoding RNAs (lncRNAs) and messenger RNAs (mRNAs)

Type	Gene	Maximum Cq	Median Cq	% expression	BestKeeper r factor	**BestKeeper r**^**2**^**factor**	BestKeeper Ranking	NormFinder Stability value	NormFinder Ranking	geNorm M value	geNorm Ranking
lncRNAs	BISPR	31.4	28.7	100.0	0.833	0.694	20	0.084	22	0.416	18
	CFAP58-DT	32.6	28.6	100.0	0.712	0.508	27	0.088	24	0.472	25
	DAPK1–IT1	32.6	29.5	100.0	0.794	0.630	23	0.074	18	0.460	24
	DLEU1	31.1	27.5	100.0	0.887	0.787	7	0.040	3	0.368	11
	DLEU2	29.5	26.3	100.0	0.827	0.685	21	0.054	7	0.409	17
	EGOT	32.6	29.5	100.0	0.862	0.743	12	0.073	17	0.390	14
	EPB41L4A-AS1	30.0	27.4	100.0	0.846	0.715	17	0.086	23	0.297	5
	FAS-AS1	31.9	27.9	100.0	0.851	0.724	14	0.068	15	0.423	19
	**FGD5–AS1**	**29.0**	**25.6**	**100.0**	**0.933**	**0.871**	**2**	**0.032**	**2**	**0.013**	**2**
	FOXN3–AS2	31.4	27.7	100.0	0.864	0.746	11	0.056	8	0.430	20
	GAS5	28.0	24.9	100.0	0.848	0.720	15	0.061	13	0.376	12
	HOTAIRM1	31.1	28.2	100.0	0.563	0.317	28	0.126	29	0.522	28
	KCNQ1OT1	32.2	28.9	100.0	0.839	0.703	18	0.060	12	0.403	16
	LINC00667	32.3	28.5	100.0	0.836	0.699	19	0.096	26	0.436	21
	LUCAT1	28.5	25.0	100.0	0.772	0.596	24	0.068	16	0.451	23
	MALAT1	31.6	28.0	100.0	0.899	0.808	6	0.057	9	0.349	9
	MIAT	31.4	27.1	100.0	0.765	0.586	25	0.123	28	0.502	27
	MIR3142HG	32.6	28.8	100.0	0.881	0.776	9	0.079	20	0.397	15
	OIP5–AS1	29.4	26.1	100.0	0.808	0.653	22	0.081	21	0.442	22
	SNHG1	32.1	28.9	100.0	0.727	0.529	26	0.089	25	0.485	26
	SNHG20	32.8	28.8	100.0	0.924	0.854	4	0.058	10	0.333	8
	SNHG5	27.5	25.0	100.0	0.543	0.294	29	0.104	27	0.544	29
	SNHG6	26.9	24.7	100.0	0.846	0.716	16	0.077	19	0.289	4
	SNHG7	31.3	27.3	100.0	0.854	0.729	13	0.066	14	0.382	13
	THRIL	29.6	27.0	100.0	0.867	0.751	10	0.051	5	0.304	6
	TSIX	32.3	28.7	100.0	0.920	0.847	5	0.059	11	0.360	10
	TSPOAP1–AS1	28.0	25.9	100.0	0.884	0.781	8	0.052	6	0.315	7
	**TUG1**	**28.5**	**25.2**	**100.0**	**0.981**	**0.963**	**1**	**0.026**	**1**	**0.013**	**2**
	**ZFAS1**	**27.1**	**24.2**	**100.0**	**0.928**	**0.861**	**3**	**0.049**	**4**	**0.241**	**3**
mRNAs	ACTB	23.53	20.65	100.0	0.881	0.776	7	0.058	6	0.329	7
	**B2M**	**19.83**	**17.33**	**100.0**	**0.951**	**0.905**	**3**	**0.049**	**3**	**0.271**	**5**
	GUS	31.18	27.56	100.0	0.941	0.885	4	0.050	4	0.224	3
	**HPRT1**	**31.47**	**28.13**	**100.0**	**0.966**	**0.933**	**1**	**0.029**	**2**	**0.012**	**2**
	**PPIA**	**25.88**	**22.94**	**100.0**	**0.965**	**0.931**	**2**	**0.027**	**1**	**0.012**	**2**
	RPLP0	25.34	22.44	100.0	0.906	0.821	6	0.074	7	0.290	6
	TBP	31.76	28.89	100.0	0.912	0.832	5	0.052	5	0.249	4
	TFCR	28.14	25.25	100.0	0.632	0.399	8	0.093	8	0.410	8

Independent stability rankings of candidate lncRNA and mRNA reference genes according to three algorithms commonly used for reference gene validation. geNorm provides an *M* value based on the average pairwise variation between genes; NormFinder estimates a stability value by modeling both intra- and inter-group variation; and BestKeeper calculates a correlation coefficient (r) with the BestKeeper index, based on pairwise correlations and the standard deviation of Cq values. Lower values indicate higher stability for geNorm and NormFinder, whereas higher correlation coefficients indicate greater stability for BestKeeper

To assess their suitability, we normalized the lncRNA dataset using several strategies and compared the residual variability to that of the not normalized data (Fig. 1B). Mean-centering produced the greatest reduction, consistent with the observations of Mestdagh et al. [12] in microRNA profiling, where the geometric mean of selected candidates closely approximated the mean of the entire dataset. Strikingly, TUG1 alone outperformed multiple reference combinations in reducing variability, achieving a variability reduction nearly equivalent to that of mean-centering. We also tested eight widely used mRNA reference genes included in the assay (Supplemental Table S1). Applying the same selection criteria, the leading candidates were HPRT1/PPIA (geNorm), HPRT1 (NormFinder) and PPIA (BestKeeper), with B2M added as a third candidate (Fig. 1C and Table 1). None of the mRNA-based normalization approaches, whether single or combined, matched the performance of mean-centering and all were clearly outperformed by TUG1 (Fig. 1D). A direct comparison of stability, including both lncRNA and classical mRNA reference genes, further confirmed that TUG1 remained among the most stable candidates (Supplemental Figure S1A and Table S4) and achieved the greatest reduction in variability (Supplemental Figure S1B). Moreover, we assessed whether TUG1 expression varied with clinical characteristics of the cohort. No meaningful associations were detected (Supplemental Table S5), supporting its stability across patient subgroups. Finally, TUG1 consistently ranked among the most highly expressed and stable lncRNAs across four external RNA-seq datasets, including samples from healthy individuals and patients with amyotrophic lateral sclerosis, acute myocardial infarction and Long COVID (Supplemental Figure S2).

In summary, TUG1 is a potential endogenous reference gene for lncRNA quantification in blood-based studies. Its outstanding performance positions it as a promising and cost-effective candidate to ensure accurate and reproducible results in translational research settings, being faster, simpler and less computationally demanding than mean-centering, thus facilitating its direct implementation in routine clinical workflows.

The strengths of this study include a well-characterized cohort, large sample size and broad lncRNA panel enabling robust and unbiased candidate evaluation. Limitations include the focus on a single, highly specific patient population (COVID-ARDS survivors) and the use of a restricted lncRNA panel. Therefore, validation of TUG1 as a reference gene in independent cohorts and samples collected in different centers, is required to confirm its robustness and general applicability. Moreover, the consideration of reference lncRNAs from diverse functional categories would help minimize co-regulation risk.

## Electronic supplementary material

Below is the link to the electronic supplementary material.

Supplementary Material 1

## Data Availability

The datasets generated and/or analyzed during the current study are available from the corresponding author on reasonable request.
