# Supplementary material for: TUG1: a potential endogenous reference gene for long noncoding RNA quantification in blood-based studies
Source: Biomark Res. 2025 Dec 30;13:161. doi: 10.1186/s40364-025-00871-2 (PMC12754918; doi:10.1186/s40364-025-00871-2)
Supplement: Supplementary file 1 — Supplementary Material 1 [file 40364_2025_871_MOESM1_ESM.docx]

**Title.** TUG1: a potential endogenous reference gene for long noncoding RNA quantification in blood-based studies.

**Short title**. Endogenous reference lncRNAs in whole blood.

Carlos Rodríguez-Muñoz,^1,2^ Anna Vila,^1^ Sally Santisteve,^1,2^ Anna Sánchez-Cucó,^1^ Iván D Benítez,^1,2,3^ María C García-Hidalgo,^1,2^ Marta Molinero,^1,2^ Manel Perez-Pons,^1,2^ Anna Moncusí-Moix,^1,2^ Ferran Barbé,^1,2^ Jessica González,^1,2,*^ David de Gonzalo-Calvo,^1,2,*^

1. Translational Research in Respiratory Medicine, University Hospital Arnau de Vilanova and Santa Maria, IRBLleida, Lleida, Spain.
2. CIBER of Respiratory Diseases (CIBERES), Institute of Health Carlos III, Madrid, Spain.
3. Department of Basic Medical Sciences, Lleida Biomedical Research Institute (IRBLleida), University of Lleida, Lleida, Spain.

* equally contributed

**Corresponding author:**

David de Gonzalo-Calvo, PhD

Translational Research in Respiratory Medicine, University Hospital Arnau de Vilanova and Santa Maria, IRBLleida.

Avda, Alcalde Rovira Roure 80 · 25198 Lleida, Spain.

Tel: +34 973702491 / E-mail: [dgonzalo@irblleida.cat](mailto:dgonzalo@irblleida.cat)

**SUPPLEMENTARY METHODS**

**Study design and population**

This is a substudy of the CIBERESUCICOVID study, registered at [www.clinicaltrials.gov](http://www.clinicaltrials.gov) under identifier NCT04457505 [1]. This prospective study included 182 patients with severe COVID-19 requiring mechanical ventilation, admitted to the Hospital Universitari Arnau de Vilanova-Santa María (Lleida, Spain). Patients were eligible if they met the following inclusion criteria: aged over 18, developed ARDS secondary to SARS-CoV-2 infection during the hospital stay according to the Berlin definition [2], required invasive mechanical ventilation (IMV) during de intensive care unit (ICU) stay and attended a “Post-COVID” evaluation after hospital discharge (median [P25; P75] of 3.95 [3.36; 5.02] months). Exclusion criteria included: death during follow-up, transfer to another department or institution, incomplete follow-up or presence of non-COVID-related conditions that could interfere with follow-up or affect respiratory function tests.

Demographic, clinical, pharmacological and laboratory data were extracted from the electronic medical records and recorded in a REDCap database.

**Sample collection**

Samples were processed under standardized conditions with the support of the IRBLleida Biobank (B.0000682) and Biobank and Biomodels Platform ISCIII PT23/00032. Venous blood was collected after an overnight fasting using Tempus™ Blood RNA tubes (Thermo Fisher Scientific), in accordance with the manufacturer’s instructions. All samples were stored at −80 °C until analysis.

The Tempus™ Whole Blood RNA isolation system offers an alternative approach to peripheral blood RNA isolation. Tempus™ Blood RNA Tubes are suitable for stabilization and isolation of total RNA from whole blood for gene expression analysis. The tubes contain 6 mL of a stabilizing reagent that lyses whole blood cells immediately after collection and inactivates cellular RNases.

**RNA Isolation**

RNA isolation, purification and quantification were performed by experienced personnel blinded to clinical data, following standardized protocols as previously described [3].

Briefly, total RNA was isolated from 3 mL of preprocessed whole blood with the Maxwell® RSC simply RNA Blood Kit (Promega) in conjunction with the Maxwell® RSC Instruments, according to the manufacturer's protocol. This RNA isolation kit is designed for isolation of total RNA from whole blood with minimal sample handling before automated purification on the Maxwell® Instruments. The Maxwell® RSC simply RNA Blood Kit purifies samples using a paramagnetic particle that provides a mobile solid phase that optimizes sample capture, washing and purification of nucleic acid. The magnetic particle-handling instruments efficiently bind RNA to the paramagnetic particle in the first well of a prefilled cartridge. The samples are processed through a series of washes before the RNA is eluted. The low elution volume results in concentrated high-quality RNA suitable for use in downstream applications such as quantitative real-time polymerase chain reaction (qPCR).

To further enhance RNA yield and purity, samples were subsequently processed with the RNeasy MinElute Cleanup Kit (Qiagen), also following manufacturer recommendations. RNeasy MinElute technology combines the selective binding properties of a silica-based membrane with the speed of microspin technology. Guanidine-thiocyanate–containing lysis buffer and ethanol are added to the sample to promote selective binding of RNA to the silica membrane of the RNeasy MinElute spin column. The sample is then applied to the RNeasy MinElute spin column. RNA binds to the silica membrane, contaminants are efficiently washed away and high-quality RNA is eluted in RNase-free water. With the RNeasy MinElute procedure, all RNA molecules longer than 200 nucleotides are purified. The kit allows minimal elution volumes, making it well-suited for concentration of RNA.

RNA concentration and purity were assessed using IMPLEN NanoPhotometer® N60 spectrophotometer (IMPLEN) by independent technical staff.

**RT-qPCR**

Reverse transcription (RT) was performed using the StaRT Reverse Transcription Kit (AnyGenes) according to the manufacturer’s protocol. First, 500 ng of total RNA in a maximum volume of 10 μL were incubated for 5 minutes at 65 °C to denature the RNA. Then, 10 μL of pre-prepared StaRT Reaction Mix (1 μL of StaRT Reverse Transcriptase 50 U/μL, 2 μL of StaRT Buffer 10×, 2 μL of Random Primers 10×, 2 μL of dNTP Mix 10× and 3 μL of ultra-pure H₂O) were added to the template to reach a final volume of 20 μL. No-template controls and no-reverse transcriptase controls were included. RT was performed under the following conditions: 10 minutes at 25 °C, 120 minutes at 37 °C, 5 minutes at 85 °C, followed by a final hold at 4 °C. Resulting cDNA samples were stored at −20 °C until use.

Long noncoding RNAs (lncRNAs) were quantified by RT-qPCR using Perfect Master Mix (AnyGenes), following the manufacturer’s protocol. The cDNA samples were diluted 1:6 prior to analysis. Quantification was performed using the “Human lncRNAs and Inflammatory & Immune Response” qPCR SignArrays® 384 system (AnyGenes), which contains predesigned and pre-coated primers for 84 lncRNAs and 8 literature-supported mRNA reference genes **(Supplemental Table S1)**. AnyGenes specifically designs and validates its primers under strict quality control procedures, including intron-spanning design whenever possible, single-peak melting curves and verified amplification efficiency within 90–110%. The lncRNA panel was selected from a group of transcripts that are among the best characterized in the literature, many of which have been described in immune- and inflammation-related contexts. However, given the pleiotropic nature of lncRNAs, these molecules often participate in multiple biological pathways beyond their initial functional annotation. This strategy allowed us to focus on well-established candidates with broad biological relevance. For each well, the reaction mixture consisted of 1 μL of 1:6 diluted cDNA, 5 μL of 2× Perfect Master Mix SYBR Green and 4 μL of ultra-pure H_2_O, for a total volume of 10 μL. After loading, plates were centrifuged and subjected to qPCR using the QuantStudio™ 7 Flex Real-Time PCR System (Applied Biosystems) under the following conditions: 10 minutes at 95 °C, followed by 40 cycles of 10 seconds at 95 °C and 30 seconds at 60 °C. A melting curve analysis was then performed: 10 seconds at 95 °C followed by a gradual increase from 65 °C to 95 °C. Negative and positive controls were included in each plate. Positive controls served both as interplate calibrators and quality controls for qPCR performance.

Amplification curves were analyzed using QuantStudio Software v1.3 (Thermo Fisher Scientific). Melting curves were examined to confirm the presence of single amplification products and absence of primer dimers. The quantification cycle (Cq) was defined as the fractional cycle number at which fluorescence exceeded the threshold.

**Stability analysis and evaluation of endogenous reference genes**

Candidate lncRNAs that meet the expression criteria: detected in 100% of samples, with a maximum Cq < 33 and a median Cq < 30, were selected for further stability analysis. These lncRNAs were evaluated using three widely recognized algorithms: geNorm, NormFinder and BestKeeper [4–6]. Data preprocessing and analysis were conducted according to each algorithm’s original specifications.

In the geNorm algorithm, the M value is defined as the average pairwise variation between a given candidate and all other genes in the dataset; lower M values indicate higher expression stability. The algorithm iteratively removes the least stable gene and recalculates M values, ultimately identifying the two most stable reference genes [4]. In NormFinder, a model-based approach estimates intra- and intergroup variation for each candidate gene. It then calculates a stability value that reflects the combined expression variance within and between groups, with lower values indicating more stable expression [5]. BestKeeper calculates the geometric mean of the most highly correlated gene pairs to create a BestKeeper index. Each candidate gene is then correlated with this index using Pearson correlation coefficients (r) and coefficient of determination (r^2^), where higher values indicate greater stability [6].

A candidate lncRNA was selected as a reference gene if it fulfilled at least one of the following criteria: i) ranked first in any of the three algorithms; or ii) ranked within the top three in at least two algorithms. These criteria were chosen to identify multiple high-confidence candidates, as the use of more than one endogenous control is generally recommended for accurate normalization [7].

The relative expression of each lncRNA was normalized using either the mean-centering strategy or the selected reference genes by subtracting the reference value from each individual expression value. When mean-centering was applied, the geometric mean of all lncRNAs meeting the expression criteria was calculated for each sample and used as a single normalization factor. The same procedure was followed when two or more reference genes were used; their geometric mean was calculated per sample and employed as a single normalization factor. When a single reference gene was used, its expression value for each sample served as the normalization factor. After the normalization process, the coefficient of variation (CV) for each normalization strategy (mean-centering and candidate reference genes) was calculated as the ratio of the standard deviation to the mean of the normalized expression values. Finally, the cumulative distribution of CV values across all lncRNAs was analyzed to compare the performance of different normalization methods in reducing variability.

All stability analyses were conducted using R statistical software, version 4.4.2 (R Project for Statistical Computing).

**Validation of selected lncRNA candidate in external datasets**

Bioinformatic analysis was performed using R software, version 4.4.2 (R Project for Statistical Computing, [www.r-project.org](http://www.r-project.org/)). Data from the Gene Expression Omnibus (GEO) database in NCBI (<https://www.ncbi.nlm.nih.gov/geo/>) [8] were processed using the DESeq2 package [9]. Four different RNA sequencing (RNA-seq) datasets from whole blood samples were selected for analysis: GSE234297, GSE218474, GSE270454 and GSE227116. These datasets included samples from healthy individuals as well as patients with amyotrophic lateral sclerosis, acute myocardial infarction and Long COVID.

For each dataset, the top quartile of lncRNAs based on mean expression levels was identified. The variability of these lncRNAs was then assessed by calculating the CV and the position of TUG1 within each CV ranking was determined to evaluate its relative stability within independent cohorts.

**Statistical analysis**

Descriptive statistics were used to summarize the characteristics of the study population. Data are presented as the median [P25; P75] for continuous variables and as frequencies (percentage) for categorical variables. Group comparisons of lncRNA expression according to clinical variables were performed using nonparametric tests (Mann–Whitney U for two groups, Kruskal–Wallis for multiple groups). Correlations between continuous variables were assessed with Spearman’s rank correlation coefficient. A p-value < 0.05 was considered statistically significant.

All analyses were conducted in R software, version 4.4.2 (R Project for Statistical Computing).

**References**

1. Torres A, Arguimbau M, Bermejo-Martín J, Campo R, Ceccato A, Fernandez-Barat L, et al. CIBERESUCICOVID: un proyecto estratégico para una mejor comprensión y manejo clínico de la COVID-19 en pacientes críticos. Arch Bronconeumol. 2021;57:1–2.

2. Ranieri VM, Rubenfeld GD, Thompson BT, Ferguson ND, Caldwell E, Fan E, et al. Acute Respiratory Distress Syndrome. JAMA. 2012;307(23):2526–33.

3. García-Hidalgo MC, Peláez R, González J, Santisteve S, Benítez ID, Molinero M, et al. Genome-wide transcriptional profiling of pulmonary functional sequelae in ARDS- secondary to SARS-CoV-2 infection. Biomed Pharmacother. 2022;154:113617.

4. Vandesompele J, De Preter K, Pattyn F, Poppe B, Van Roy N, De Paepe A, et al. Accurate normalization of real-time quantitative RT-PCR data by geometric averaging of multiple internal control genes. Genome Biol. 2002;3(7):research0034.1.

5. Andersen CL, Jensen JL, Ørntoft TF. Normalization of Real-Time Quantitative Reverse Transcription-PCR Data: A Model-Based Variance Estimation Approach to Identify Genes Suited for Normalization, Applied to Bladder and Colon Cancer Data Sets. Cancer Res. 2004;64(15):5245–50.

6. Pfaffl MW, Tichopad A, Prgomet C, Neuvians TP. Determination of stable housekeeping genes, differentially regulated target genes and sample integrity: BestKeeper – Excel-based tool using pair-wise correlations. Biotechnol Lett. 2004;26:509–15.

7. Schwarzenbach H, da Silva AM, Calin G, Pantel K. Data Normalization Strategies for MicroRNA Quantification. Clin Chem. 2015;61(11):1333–42.

8. Barrett T, Wilhite SE, Ledoux P, Evangelista C, Kim IF, Tomashevsky M, et al. NCBI GEO: archive for functional genomics data sets—update. Nucleic Acids Res. 2013;41(D1):D991–5.

9. Love MI, Huber W, Anders S. Moderated estimation of fold change and dispersion for RNA-seq data with DESeq2. Genome Biol. 2014;15(12):1–21.

**SUPPLEMENTARY FIGURES**


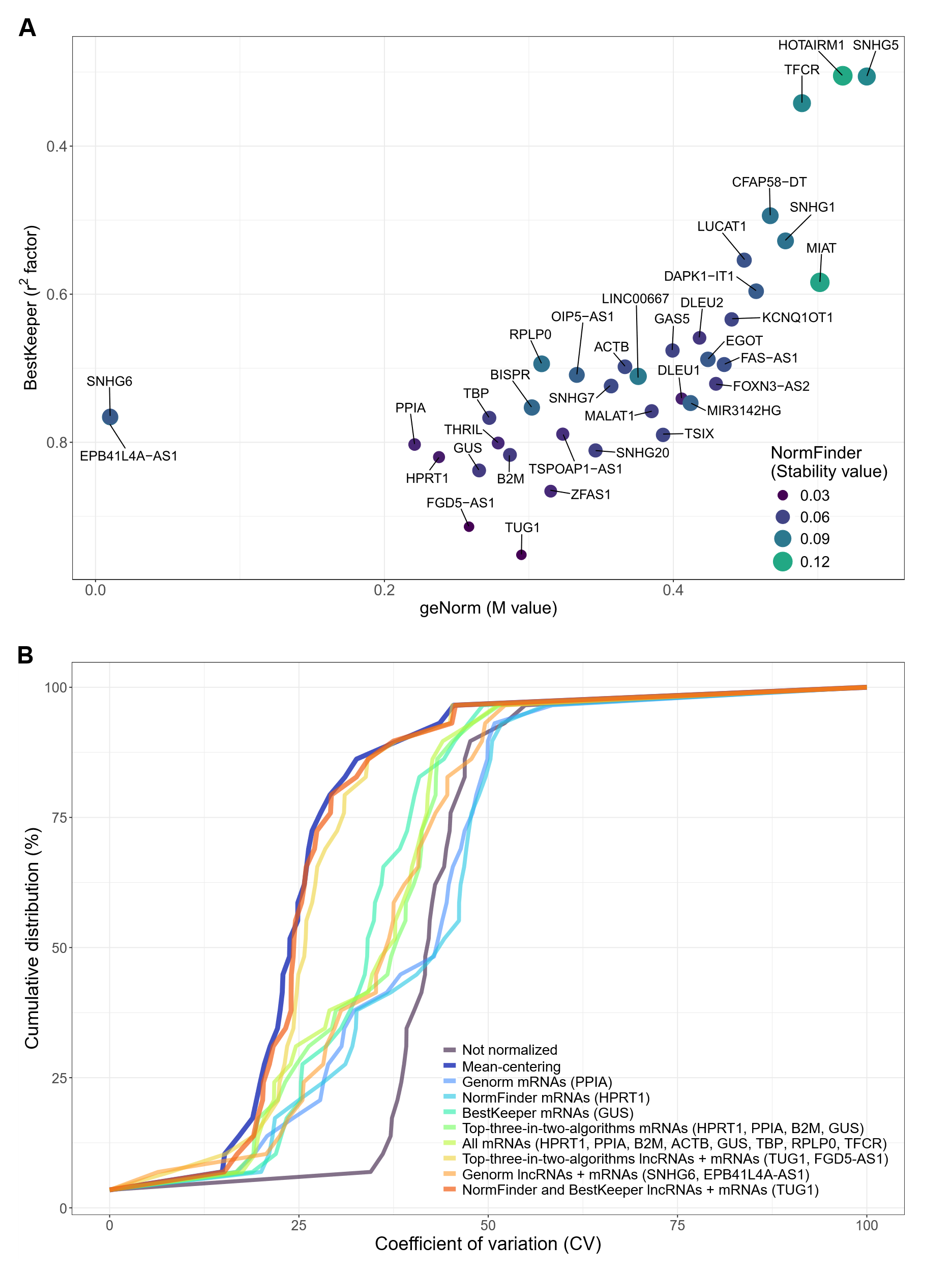


**Supplemental Figure S1. Stability and suitability of candidate endogenous reference long noncoding RNAs (lncRNAs) and messenger RNAs (mRNAs) from the joint analysis of all candidates.** A) Stability values for each candidate lncRNA and mRNA were calculated using three commonly applied algorithms: geNorm, NormFinder and BestKeeper. B) Cumulative distribution plots of the coefficient of variation (CV) for lncRNA expression data under different normalization strategies: unnormalized data, mean-centering the best-ranked candidate from each algorithm, the top three candidates ranked in at least two algorithms, the best-ranked mRNA from each algorithm and the top three mRNAs ranked in at least two algorithms.

**
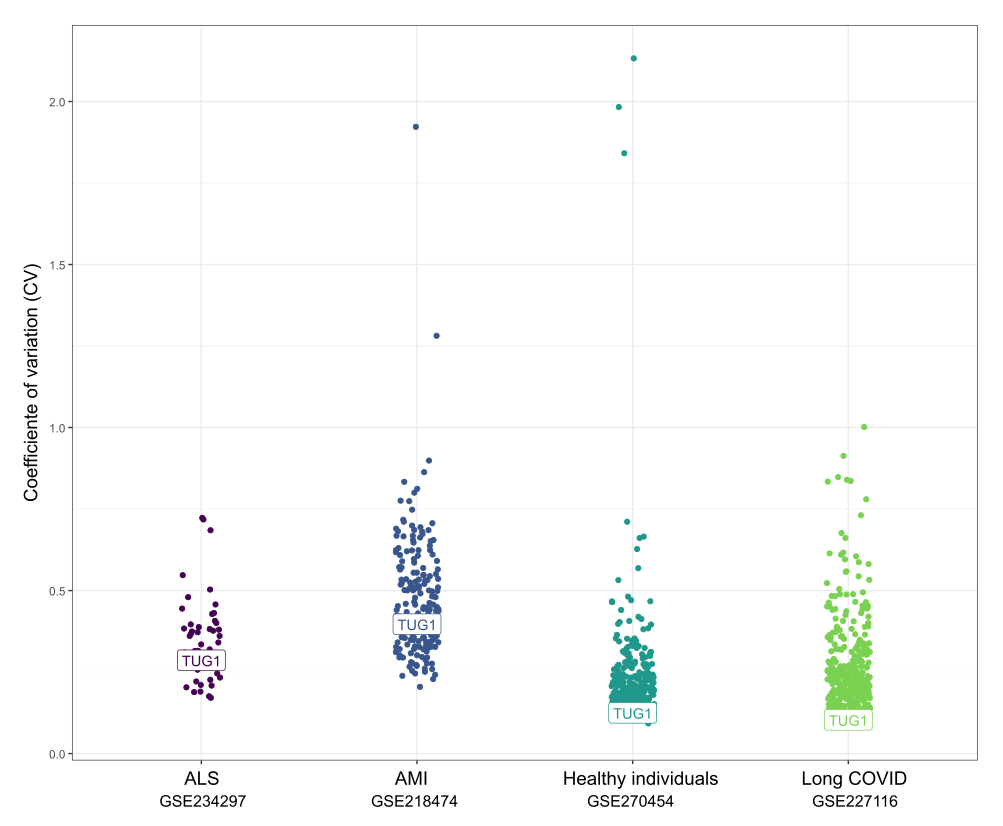
**

**Supplemental Figure S2. Variability of TUG1 across four external RNA-seq datasets.** Long noncoding RNAs (lncRNAs) within the top quartile of expression were analyzed across four independent RNA-seq datasets from whole blood samples obtained from healthy individuals (GSE270454) and patients with different clinical conditions: amyotrophic lateral sclerosis (ALS, GSE234297), acute myocardial infarction (AMI, GSE218474) and Long COVID (GSE227116). The plot shows the coefficients of variation (CV) of lncRNAs within the top quartile of expression for each dataset. The relative position of TUG1, labeled with its name, is indicated within each distribution. TUG1 consistently ranked among the most highly expressed and least variable lncRNAs across all four cohorts, supporting its stability in independent populations and disease contexts.

**SUPPLEMENTARY TABLES**

**Supplemental Table S1. List of long noncoding RNAs and messenger RNAs.**

|  | Symbol | Gene name | RefSeq | Ch. Location | Forward Primer location | Reverse Primer location | Amplicon size (bp) |
| --- | --- | --- | --- | --- | --- | --- | --- |
| Long non-coding RNAs | **A2ML1-AS1** | A2ML1 antisense RNA 1 | ENST00000537288.1 | 12p13.31 | exon 1 | exon 1 | 101 |
|  | **BISPR** | BST2 interferon stimulated positive regulator | NR_130765.1 | 19p13.11 | exon 4 | exon 5 | 87 |
|  | **CASC2** | cancer susceptibility 2 | NR_026939.1 | 10q26.11 | exon 2 | exon 3 | 148 |
|  | **CCAT1** | colon cancer associated transcript 1 | NR_108049.1 | 8q24.21 | Junction 1-2 | exon 2 | 159 |
|  | **CDKN2B-AS1** | CDKN2B antisense RNA 1 | NR_003529.3 | 9p21.3 | exon 1 | exon 1 | 133 |
|  | **CFAP58-DT** | CFAP58 divergent transcript | NR_108036.1 | 10q25.1 | exon 1 | exon 1 | 129 |
|  | **DAPK1-IT1** | DAPK1 intronic transcript 1 | NR_146781.1 | 9q21.33 | exon 2 | exon 2 | 117 |
|  | **DLEU1** | deleted in lymphocytic leukemia 1 | NR_109973.1 | 13q14.2-q14.3 | exon 1 | exon 1 | 142 |
|  | **DLEU2** | deleted in lymphocytic leukemia 2 | NR_002612.1 | 13q14.3 | exon 12 | exon 12 | 120 |
|  | **EGOT** | eosinophil granule ontogeny transcript | NR_004428.1 | 3p26.1 | exon 1 | exon 1 | 130 |
|  | **EMX2OS** | EMX2 opposite strand/antisense RNA | NR_002791.2 | 10q26.1 | exon 4 | exon 4 | 119 |
|  | **EPB41L4A-AS1** | EPB41L4A antisense RNA 1 | NR_015370.2 | 5q22.1 | exon 1 | exon 2 | 133 |
|  | **FAM30A** | family with sequence similarity 30 member A | NR_026800.2 | 14q32.33 | exon 6 | exon 6 | 122 |
|  | **FAS-AS1** | FAS antisense RNA 1 | NR_028371.1 | 10q24.1 | exon 1 | exon 1 | 138 |
|  | **FGD5-AS1** | FGD5 antisense RNA 1 | NR_046251.1 | 3p25.1 | exon 2 | exon 2 | 145 |
|  | **FGF14-IT1** | FGF14 intronic transcript 1 | NR_036486.1 | 13q33.1 | exon 4 | exon 4 | 127 |
|  | **FOXN3-AS2** | FOXN3 antisense RNA 2 | NR_024620.1 | 14q32.11 | exon 1 | exon 1 | 110 |
|  | **GAS5** | growth arrest specific 5 | NR_002578.2 | 1q25.1 | Junction 11-12 | exon 12 | 82 |
|  | **GAS5-AS1** | GAS5 antisense RNA 1 | NR_037605.1 | 1q25.1 | exon 1 | exon 1 | 136 |
|  | **GNAS-AS1** | GNAS antisense RNA 1 | NR_002785.2 | 20q13.32 | exon 3 | exon 4 | 117 |
|  | **GRM5-AS1** | GRM5 antisense RNA 1 | NR_049724.1 | 11q14.2 | exon 5 | exon 6 | 112 |
|  | **H19** | H19 imprinted maternally expressed transcript | NR_002196.2 | 11p15.5 | exon 2 | exon 3 | 138 |
|  | **HIF1A-AS1** | HIF1A antisense RNA 1 | NR_047116.1 | 14q23.2 | exon 2 | exon 2 | 90 |
|  | **HOTAIR** | HOX transcript antisense RNA | NR_003716.3 | 12q13.13 | exon 4 | exon 5 | 144 |
|  | **HOTAIRM1** | HOXA transcript antisense RNA, myeloid-specific 1 | NR_038367.1 | 7p15.2 | exon 2 | exon 2 | 93 |
|  | **HOTTIP** | HOXA distal transcript antisense RNA | NR_037843.3 | 7p15.2 | exon 2 | exon 2 | 85 |
|  | **HOXA11-AS** | HOXA11 antisense RNA | NR_002795.2 | 7p15.2 | exon 2 | exon 2 | 73 |
|  | **HOXA-AS2** | HOXA cluster antisense RNA 2 | NR_122069.1 | 7p15.2 | exon 1 | exon 2 | 91 |
|  | **HOXA-AS3** | HOXA cluster antisense RNA 3 | NR_038831.1 | 7p15.2 | exon 2 | exon 2 | 97 |
|  | **HULC** | hepatocellular carcinoma up-regulated long non-coding RNA | NR_004855.2 | 6p24.3 | exon 1 | exon 1 | 130 |
|  | **IFNG-AS1** | IFNG antisense RNA 1 | NR_104124.1 | 12q15 | exon 4 | exon 4 | 154 |
|  | **JPX** | JPX transcript, XIST activator | NR_024582.1 | Xq13.2 | exon 5 | exon 5 | 114 |
|  | **KCNQ1OT1** | KCNQ1 opposite strand/antisense transcript 1 | NR_002728.3 | 11p15 | exon 1 | exon 1 | 93 |
|  | **LINC00305** | long intergenic non-protein coding RNA 305 | NR_027245.1 | 18q22.1 | exon 1 | exon 1 | 99 |
|  | **LINC00511** | long intergenic non-protein coding RNA 511 | NR_033876.1 | 17q24.3 | exon 4 | exon 4 | 73 |
|  | **LINC00525** | long intergenic non-protein coding RNA 525 | NR_038407.1 | 7p12.3 | Junction 1-2 | exon 2 | 104 |
|  | **LINC00635** | long intergenic non-protein coding RNA 635 | NR_015414.1 | 3q13.12 | exon 3 | exon 4 | 89 |
|  | **LINC00667** | long intergenic non-protein coding RNA 667 | NR_015389.1 | 18p11.31 | exon 3 | exon 3 | 126 |
|  | **LINC00930** | long intergenic non-protein coding RNA 930 | NR_021493.1 | 15q26.1 | exon 1 | exon 1 | 122 |
|  | **LINC01991** | long intergenic non-protein coding RNA 1991 | NR_135537.1 | 3q27.3 | exon 1 | Junction 1-2 | 89 |
|  | **lnc-IL7R-1:1** | novel transcript/ LNCipedia transcript ID: lnc-IL7R-1:1 | ENST00000503269 | chr5 | exon 2 | exon 2 | 69 |
|  | **LncRNA-ATB** | long noncoding RNA activated by TGF-beta | NR_160525.1 | 14q11.2 | exon 1 | exon 1 | 92 |
|  | **LUCAT1** | lung cancer associated transcript 1 (non-protein coding) | NR_103548.1 | 5q14.3 | exon 1 | exon 1 | 120 |
|  | **LUNAR1** | leukemia-associated non-coding IGF1R activator RNA 1 | NR_126487.1 | 15q26.3 | exon 3 | exon 3 | 129 |
|  | **MAFTRR** | MAF transcriptional regulator RNA | NR_104663.1 | 16q23.2 | exon 2 | exon 3 | 97 |
|  | **MAILR** | macrophage interferon regulatory lncRNA | NR_126338.1 | 8q22.3 | exon 4 | exon 4 | 136 |
|  | **MALAT1** | metastasis associated lung adenocarcinoma transcript 1 | NR_002819.3 | 11q13.1 | exon 1 | exon 1 | 87 |
|  | **MEG3** | maternally expressed 3 | NR_003530.2 | 14q32 | exon 7 | exon 8 | 67 |
|  | **MIAT** | myocardial infarction associated transcript | NR_003491.3 | 22q12.1 | exon 3 | exon 3 | 131 |
|  | **MIR3142HG** | MIR3142 host gene | NR_132748.1 | 5q33.3 | exon 2 | exon 2 | 121 |
|  | **MIR31HG** | MIR31 host gene | NR_027054.2 | 9p21.3 | exon 4 | exon 4 | 90 |
|  | **MROCKI** | MARCKS cis regulating lncRNA promoter of cytokines and inflammation | NR_038863.2 | 6q21 | exon 1 | Junction 1-2 | 118 |
|  | **NALT1** | NOTCH1 associated lncRNA in T cell acute lymphoblastic leukemia 1 | NR_121577.1 | 9q34.3 | exon 1 | exon 1 | 76 |
|  | **NEAT1** | nuclear paraspeckle assembly transcript 1 | NR_131012.1 | 11q13.1 | exon 1 | exon 1 | 147 |
|  | **NFIA-AS1** | NFIA antisense RNA 1 | NR_104180.1 | 1p31.3 | Junction 3-4 | exon 4 | 135 |
|  | **NKILA** | NF-kappaB interacting lncRNA | NR_131157.1 | 20q13.31 | exon 1 | exon 1 | 129 |
|  | **NRAV** | negative regulator of antiviral response | NR_038854.1 | 12q24.31 | exon 2 | exon 2 | 116 |
|  | **NRIR** | negative regulator of interferon response | NR_126359.1 | 2p25.2 | exon 1 | Junction 1-2 | 149 |
|  | **NRON** | non-coding repressor of NFAT | NR_045006.1 | 9q33.3; 9 | exon 1 | exon 1 | 97 |
|  | **OIP5-AS1** | OIP5 antisense RNA 1 | NR_152822.1 | 15q15.1 | Junction 3- 4 | exon 4 | 105 |
|  | **PACERR** | PTGS2 antisense NFKB1 complex-mediated expression regulator RNA | NR_125801.1 | 1q31.1 | exon 1 | exon 1 | 99 |
|  | **PANTR1** | POU3F3 adjacent non-coding transcript 1 | NR_037883.1 | 2q12.1 | Junction 2-3 | exon 3 | 150 |
|  | **PCAT1** | prostate cancer associated transcript 1 | NR_045262.1 | 8q24.21 | exon 2 | exon 2 | 117 |
|  | **PVT1** | Pvt1 oncogene | NR_003367.3 | 8q24 | exon 5 | exon 6 | 115 |
|  | **ROR1-AS1** | ROR1 antisense RNA 1 | NR_110665.1 | 1p31.3 | exon 3 | exon 3 | 135 |
|  | **SALRNA1** | senescence associated long non-coding RNA 1 | NR_126480.1 | 14q23.1 | exon 1 | exon 1 | 160 |
|  | **SBF2-AS1** | SBF2 antisense RNA 1 | NR_036485.1 | 11p15.4 | exon 1 | exon 2 | 109 |
|  | **SLC7A11-AS1** | SLC7A11 antisense RNA 1 | NR_038380.1 | 4q28.3 | exon 7 | exon 7 | 136 |
|  | **SNHG1** | small nucleolar RNA host gene 1 | NR_003098.1 | 11q12.3 | exon 7 | exon 9 | 90 |
|  | **SNHG16** | small nucleolar RNA host gene 16 | NR_038108.1 | 17q25.1 | exon 3 | exon 3 | 140 |
|  | **SNHG20** | small nucleolar RNA host gene 20 | NR_027058.1 | 17q25.2 | exon 3 | exon 3 | 149 |
|  | **SNHG5** | small nucleolar RNA host gene 5 | NR_003038.2 | 6q14.3 | exon 4 | exon 5 | 107 |
|  | **SNHG6** | small nucleolar RNA host gene 6 | NR_002599.1 | 8q13; 8q13 | Junction 1-2 | exon 2 | 112 |
|  | **SNHG7** | small nucleolar RNA host gene 7 | NR_003672.2 | 9q34.3 | exon 2 | exon 2 | 91 |
|  | **SOX2-OT** | SOX2 overlapping transcript | NR_075091.1 | 3q26.33 | exon 8 | exon 8 | 114 |
|  | **THRIL** | TNF and HNRNPL related immunoregulatory long non-coding RNA | NR_110375.1 | 12q24.31 | exon 1 | exon 1 | 123 |
|  | **TMC3-AS1** | TMC3 antisense RNA 1 | NR_120365.1 | 15q25.1 | exon 6 | exon 6 | 85 |
|  | **TSIX** | TSIX transcript, XIST antisense RNA | NR_003255.2 | Xq13.2 | exon 1 | exon 1 | 124 |
|  | **TSPOAP1-AS1** | TSPOAP1, SUPT4H1 and RNF43 antisense RNA 1 | NR_038410.1 | 17q22 | exon 3 | exon 3 | 88 |
|  | **TUG1** | taurine up-regulated 1 | NR_110492.1 | 22q12.2 | exon 1 | exon 1 | 114 |
|  | **UCA1** | urothelial cancer associated 1 | NR_015379.3 | 19p13.12 | exon 2 | exon 3 | 154 |
|  | **WFDC21P** | WAP four-disulfide core domain 21, pseudogene | NR_030732.1 | 17q23.1 | exon 3 | exon 3 | 104 |
|  | **XIST** | X inactive specific transcript | NR_001564.2 | Xq13.2 | exon 4 | Junction 4-5 | 106 |
|  | **ZFAS1** | ZNFX1 antisense RNA 1 | NR_003604.2 | 20q13.13 | exon 2 | exon 2 | 93 |
| Messenger RNAs | **ACTB** | actin, beta | NM_001101 | 7p22 | 5' UTR | exon 2 | 101 |
|  | **B2M** | beta-2-microglobulin | NM_004048 | 15q21-q22.2 | junction 1-2 | exon 2 | 163 |
|  | **GUSB** | Glucuronidase, beta | NM_000181.3 | 7q11.21 | exon 4 | exon 5 | 104 |
|  | **HPRT1** | Hypoxanthine guanine phosphoribosyl transferase I | NM_000194 | Xq26.2 | exon 6 | exon 7 | 87 |
|  | **PPIA** | peptidylprolyl isomerase A (cyclophilin A) | NM_021130 | 7p13 | exon 1 | exon 2 | 97 |
|  | **RPLP0** | ribosomal protein, large, P0 | NM_053275 | 12q24.2 | exon 6 | exon 7 | 144 |
|  | **TBP** | TATA box binding protein | NM_001172085 | 6q27 | exon 5 | exon 6 | 88 |
|  | **TFRC** | Transferrin receptor (p90, CD71) | NM_001128148 | 3q26.2-qter | exon 5 | exon 6 | 87 |

**Supplemental Table S2. Characteristics of the population.**

|  | n (%) or median [P25;P75] | n |
| --- | --- | --- |
| Sociodemographic data |  |  |
| Age (years) | 62.0 [54.0;67.2] | 180 |
| Sex (female) | 46 (25.6%) | 180 |
| BMI (kg/m^2^) | 30.0 [26.9;33.1] | 180 |
| Smoking status |  | 175 |
| Never smoker | 83 (47.4%) |  |
| Former smoker | 84 (48.0%) |  |
| Current smoker | 8 (4.6%) |  |
| Time from symptoms onset to hospitalization (days) | 7.00 [5.00;9.00] | 180 |
| Comorbidities |  |  |
| Hypertension | 91 (50.6%) | 180 |
| Type II Diabetes | 52 (28.9%) | 180 |
| Obesity | 91 (50.6%) | 180 |
| Cardiovascular Disease | 17 (9.4%) | 180 |
| COPD | 7 (3.9%) | 180 |
| Asthma | 12 (6.7%) | 180 |
| Chronic renal disease | 9 (5.0%) | 180 |
| Chronic liver disease | 9 (5.0%) | 180 |
| Outcome |  |  |
| D_LCO_<80% | 137 (76.1%) | 180 |
| BMI: body mass index; COPD: chronic obstructive pulmonary disease; D_LCO_: Diffusing lung capacity for carbon monoxide. | | |

**Supplemental Table S3. Expression values of excluded lncRNA.**

| lncRNA | Maximum Cq | Median Cq | % Expression |
| --- | --- | --- | --- |
| A2ML1-AS1 | 40.29 | 36.76 | 0.00 |
| CASC2 | 40.31 | 37.19 | 0.00 |
| CCAT1 | 36.10 | 32.32 | 76.06 |
| CDKN2B-AS1 | 33.23 | 30.24 | 99.47 |
| EMX2OS | 40.00 | 35.37 | 2.66 |
| FAM30A | 40.00 | 36.26 | 0.00 |
| FGF14-IT1 | 35.05 | 32.68 | 63.30 |
| GAS5-AS1 | 33.58 | 29.45 | 99.47 |
| GNAS-AS1 | 40.00 | 34.02 | 6.91 |
| GRM5-AS1 | 39.81 | 36.02 | 0.00 |
| H19 | 34.65 | 31.50 | 85.64 |
| HIF1A-AS1 | 40.00 | 35.29 | 0.00 |
| HOTAIR | 40.00 | 35.65 | 4.26 |
| HOTTIP | 40.02 | 36.63 | 0.53 |
| HOXA11-AS | 40.14 | 40.00 | 0.00 |
| HOXA-AS2 | 39.43 | 34.58 | 2.13 |
| HOXA-AS3 | 40.00 | 35.99 | 0.53 |
| HULC | 40.00 | 33.63 | 26.06 |
| IFNG-AS1 | 33.18 | 29.30 | 99.47 |
| JPX | 36.43 | 31.36 | 97.87 |
| LINC00305 | 40.00 | 36.72 | 0.00 |
| LINC00511 | 39.67 | 34.57 | 5.32 |
| LINC00525 | 40.00 | 36.64 | 1.06 |
| LINC00635 | 34.00 | 30.79 | 98.94 |
| LINC00930 | 40.34 | 37.18 | 0.00 |
| LINC01991 | 40.27 | 40.00 | 0.53 |
| lnc-IL7R-1:1 | 40.05 | 40.00 | 0.00 |
| LncRNA-ATB | 38.80 | 34.02 | 26.60 |
| LUNAR1 | 36.89 | 30.96 | 98.40 |
| MAFTRR | 40.11 | 36.18 | 0.00 |
| MAILR | 36.72 | 31.92 | 85.11 |
| MEG3 | 36.61 | 32.38 | 72.87 |
| MIR31HG | 38.07 | 33.74 | 18.09 |
| MROCKI | 40.00 | 34.25 | 12.77 |
| NALT1 | 35.63 | 32.66 | 65.43 |
| NEAT1 | 40.00 | 22.81 | 99.47 |
| NFIA-AS1 | 40.05 | 40.00 | 0.53 |
| NKILA | 38.46 | 34.31 | 3.19 |
| NRAV | 33.22 | 30.20 | 99.47 |
| NRIR | 40.00 | 34.63 | 3.72 |
| NRON | 34.37 | 30.90 | 98.40 |
| PACERR | 37.11 | 32.57 | 71.81 |
| PANTR1 | 40.14 | 35.99 | 0.53 |
| PCAT1 | 32.98 | 30.60 | 100.00 |
| PVT1 | 33.42 | 29.83 | 99.47 |
| ROR1-AS1 | 40.00 | 37.01 | 1.60 |
| SALRNA1 | 40.22 | 39.69 | 0.00 |
| SBF2-AS1 | 34.51 | 31.61 | 96.81 |
| SLC7A11-AS1 | 39.16 | 32.86 | 56.38 |
| SNHG16 | 35.18 | 28.90 | 98.94 |
| SOX2-OT | 40.00 | 36.83 | 0.53 |
| TMC3-AS1 | 37.04 | 32.47 | 76.60 |
| UCA1 | 40.00 | 35.78 | 0.00 |
| WFDC21P | 36.33 | 31.42 | 98.94 |
| XIST | 36.34 | 33.42 | 43.09 |

**Supplemental Table S4. Expression levels, stability values and rankings from the joint stability analysis of candidate endogenous long noncoding RNAs (lncRNAs) and messenger RNAs (mRNAs).**

| **Gene** | **Maximum Cq** | **Median Cq** | **% expression** | **BestKeeper r factor** | **BestKeeper r^2^ factor** | **BestKeeper Ranking** | **NormFinder Stability value** | **NormFinder Ranking** | **geNorm *M* value** | **geNorm Ranking** |
| --- | --- | --- | --- | --- | --- | --- | --- | --- | --- | --- |
| ACTB | 23.53 | 20.65 | 100.0 | 0.836 | 0.698 | 23 | 0.064 | 20 | 0.367 | 18 |
| B2M | 19.83 | 17.34 | 100.0 | 0.904 | 0.817 | 6 | 0.056 | 10 | 0.287 | 9 |
| BISPR | 31.45 | 28.70 | 100.0 | 0.868 | 0.753 | 16 | 0.080 | 28 | 0.302 | 11 |
| CFAP58-DT | 32.59 | 28.60 | 100.0 | 0.703 | 0.494 | 34 | 0.086 | 31 | 0.467 | 32 |
| DAPK1-IT1 | 32.64 | 29.47 | 100.0 | 0.772 | 0.596 | 30 | 0.073 | 25 | 0.457 | 31 |
| DLEU1 | 31.07 | 27.46 | 100.0 | 0.861 | 0.741 | 18 | 0.043 | 4 | 0.406 | 23 |
| DLEU2 | 29.52 | 26.34 | 100.0 | 0.812 | 0.659 | 28 | 0.054 | 9 | 0.418 | 25 |
| EGOT | 32.65 | 29.47 | 100.0 | 0.829 | 0.688 | 26 | 0.072 | 24 | 0.424 | 26 |
| EPB41L4A-AS1 | **29.97** | **27.40** | **100.0** | **0.875** | **0.766** | **13** | **0.081** | **29** | **0.010** | **2** |
| FAS-AS1 | 31.86 | 27.91 | 100.0 | 0.833 | 0.695 | 24 | 0.067 | 21 | 0.435 | 28 |
| FGD5-AS1 | 28.97 | 25.62 | 100.0 | 0.956 | 0.914 | 2 | 0.029 | 2 | 0.259 | 5 |
| FOXN3-AS2 | 31.37 | 27.73 | 100.0 | 0.849 | 0.721 | 20 | 0.056 | 11 | 0.430 | 27 |
| GAS5 | 27.96 | 24.91 | 100.0 | 0.822 | 0.676 | 27 | 0.060 | 16 | 0.399 | 22 |
| GUS | 31.18 | 27.57 | 100.0 | 0.916 | 0.838 | 4 | 0.056 | 12 | 0.266 | 6 |
| HOTAIRM1 | 31.13 | 28.21 | 100.0 | 0.552 | 0.305 | 37 | 0.120 | 37 | 0.517 | 36 |
| HPRT1 | 31.47 | 28.15 | 100.0 | 0.906 | 0.820 | 5 | 0.041 | 3 | 0.238 | 4 |
| KCNQ1OT1 | 32.24 | 28.91 | 100.0 | 0.796 | 0.634 | 29 | 0.061 | 18 | 0.440 | 29 |
| LINC00667 | 32.33 | 28.48 | 100.0 | 0.843 | 0.711 | 21 | 0.091 | 33 | 0.376 | 19 |
| LUCAT1 | 28.48 | 25.03 | 100.0 | 0.744 | 0.554 | 32 | 0.068 | 22 | 0.449 | 30 |
| MALAT1 | 31.56 | 27.99 | 100.0 | 0.871 | 0.758 | 15 | 0.058 | 14 | 0.385 | 20 |
| MIAT | 31.42 | 27.07 | 100.0 | 0.764 | 0.584 | 31 | 0.117 | 36 | 0.501 | 35 |
| MIR3142HG | 32.62 | 28.76 | 100.0 | 0.865 | 0.747 | 17 | 0.077 | 27 | 0.412 | 24 |
| OIP5-AS1 | 29.38 | 26.07 | 100.0 | 0.842 | 0.709 | 22 | 0.076 | 26 | 0.333 | 15 |
| PPIA | 25.88 | 22.95 | 100.0 | 0.896 | 0.803 | 8 | 0.045 | 5 | 0.221 | 3 |
| RPLP0 | 25.34 | 22.43 | 100.0 | 0.833 | 0.694 | 25 | 0.085 | 30 | 0.309 | 12 |
| SNHG1 | 32.14 | 28.87 | 100.0 | 0.727 | 0.528 | 33 | 0.086 | 32 | 0.478 | 33 |
| SNHG20 | 32.77 | 28.77 | 100.0 | 0.900 | 0.811 | 7 | 0.059 | 15 | 0.346 | 16 |
| SNHG5 | 27.45 | 25.01 | 100.0 | 0.553 | 0.306 | 36 | 0.100 | 35 | 0.534 | 37 |
| SNHG6 | **26.89** | **24.71** | **100.0** | **0.874** | **0.765** | **14** | **0.072** | **23** | **0.010** | **2** |
| SNHG7 | 31.27 | 27.28 | 100.0 | 0.851 | 0.724 | 19 | 0.064 | 19 | 0.357 | 17 |
| TBP | 31.76 | 28.89 | 100.0 | 0.876 | 0.767 | 12 | 0.058 | 13 | 0.273 | 7 |
| TFCR | 28.14 | 25.28 | 100.0 | 0.584 | 0.342 | 35 | 0.099 | 34 | 0.489 | 34 |
| THRIL | 29.62 | 26.99 | 100.0 | 0.895 | 0.801 | 9 | 0.047 | 7 | 0.279 | 8 |
| TSIX | 32.29 | 28.72 | 100.0 | 0.889 | 0.790 | 10 | 0.061 | 17 | 0.393 | 21 |
| TSPOAP1-AS1 | 28.00 | 25.87 | 100.0 | 0.888 | 0.789 | 11 | 0.050 | 8 | 0.323 | 14 |
| TUG1 | **28.51** | **25.18** | **100.0** | **0.976** | **0.952** | **1** | **0.029** | **1** | **0.295** | **10** |
| ZFAS1 | 27.13 | 24.21 | 100.0 | 0.931 | 0.866 | 3 | 0.047 | 6 | 0.315 | 13 |

Stability ranking of candidate reference genes according to geNorm, NormFinder, and BestKeeper algorithms. Stability indices are reported as follows: M value (geNorm), stability value (NormFinder) and correlation coefficient r (BestKeeper). Lower values indicate higher stability in geNorm and NormFinder, whereas higher values indicate stronger stability in BestKeeper.

**Supplemental Table S5. Association between TUG1 levels and the clinical characteristics of the study sample.**

| Variable | | TUG1  median [P25;P75] or rho | p-value | n |
| --- | --- | --- | --- | --- |
| Sociodemographic data |  |  |  |  |
| Age (years) |  | 0.042 | 0.576 | 180 |
| Sex | Female (n=46) | 4.07 [3.83;4.22] | 0.535 | 180 |
|  | Male (n=134) | 4.08 [3.90;4.25] |  |  |
| BMI (kg/m^2^) |  | 0.015 | 0.842 | 180 |
| Smoking status* | Never smoker (n=83) | 4.01 [3.81;4.21] | 0.035 | 175 |
|  | Former smoker (n=84) | 4.13 [4.00;4.26] |  |  |
|  | Current smoker (n=8) | 3.96 [3.83;4.07] |  |  |
| Time from symptoms onset to hospitalization (days) | | -0.012 | 0.876 | 179 |
| Comorbidities |  |  |  |  |
| Hypertension | Yes (n=91) | 4.08 [3.93;4.25] | 0.665 | 180 |
|  | No (n=89) | 4.08 [3.85;4.24] |  |  |
| Type II Diabetes | Yes (n=52) | 4.11 [3.95;4.26] | 0.200 | 180 |
|  | No (n=128) | 4.06 [3.81;4.22] |  |  |
| Obesity | Yes (n=91) | 4.07 [3.85;4.22] | 0.541 | 180 |
|  | No (n=89) | 4.09 [3.89;4.25] |  |  |
| Cardiovascular Disease | Yes (n=17) | 3.98 [3.76;4.09] | 0.055 | 180 |
|  | No (n=163) | 4.09 [3.90;4.25] |  |  |
| Laboratory parameters |  |  |  |  |
| Leukocyte count (x10^9^/L) |  | 0.110 | 0.147 | 176 |
| Neutrophil count (x10^9^/L) |  | 0.101 | 0.184 | 175 |
| Lymphocyte count (x10^9^/L) |  | 0.064 | 0.415 | 167 |
| Monocyte count (x10^9^/L) |  | 0.029 | 0.704 | 176 |
| Outcome |  |  |  |  |
| D_LCO_<80% | Yes (n=137) | 4.10 [3.89;4.25] | 0.150 | 180 |
|  | No (n=43) | 4.04 [3.87;4.17] |  |  |
| BMI: body mass index; D_LCO_: Diffusing lung capacity for carbon monoxide.  * No differences when subgroups were compared. | | | | |
